# Successful Use of Fludrocortisone in a Child with Refractory Cerebral Salt Wasting Syndrome: A Case Report and Review of Literature

**DOI:** 10.7759/cureus.3505

**Published:** 2018-10-27

**Authors:** Shilpa Gurnurkar, Sindy Villacres, Lindsey Warner, Madhuradhar Chegondi

**Affiliations:** 1 Pediatrics, Nemours Children's Hospital, Orlando, USA; 2 Pediatrics, University of Central Florida College of Medicine, Orlando, USA; 3 Pediatrics, University of Iowa Stead Family Children's Hospital, Iowa City, USA

**Keywords:** cerebral salt wasting, fludrocortisone, child

## Abstract

Hyponatremia post-neurosurgical intervention can be dangerous and potentially life-threatening. Two of its most common causes are cerebral salt wasting (CSW) and syndrome of inappropriate anti-diuretic hormone release (SIADH). CSW is proposed to be secondary not only to the elevated levels of circulating atrial natriuretic peptide (ANP) and brain natriuretic peptide (BNP) but inhibition of steroidogenesis in the zona glomerulosa of the adrenal cortex, thus resulting in mineralocorticoid deficiency. We present a two-year-old male who had developed acute hyponatremia secondary to CSW on post-operative day two after a sub-total resection of a low-grade juvenile pilocytic astrocytoma (WHO grade I). Fludrocortisone was successfully used to manage the refractory hyponatremia and alleviated the need to use very large amounts of oral sodium supplementation.

## Introduction

In the intensive care and neurosurgical setting, hyponatremia is both common and concerning. A retrospective study of patients with transsphenoidal surgery for sellar tumors found a 22% prevalence of postoperative hyponatremia [1]. The estimated cost of treating hyponatremia in the US has been estimated at a minimum of 1.6 billion dollars annually [2]. More importantly, patient fatality has increased from 0.19% to 11.20% if hyponatremia (<130 mEq/L) is present when compared to matched controls [3]. The two suspected etiologies responsible for the development of non-iatrogenic hyponatremia in the intensive care unit (ICU) and in neurology ICU settings are cerebral salt wasting (CSW) and syndrome of inappropriate anti-diuretic hormone release (SIADH) [4]. The pathophysiology and management of these two conditions are very distinct, making appropriate and timely identification of these diseases crucial. Briefly, the hyponatremia associated with SIADH is dilutional due to the increased free water reabsorption. Total body sodium is unaffected, fluid status is euvolemic or hypervolemic, and total urinary salt loss is normal, though urinary salt concentration is high due to decreased free water in the urine. SIADH responds to fluid restriction [5]. Hyponatremia in CSW however, is due to true natriuresis with a high urinary salt loss, low total body salt, and hypovolemia [6]. The pathophysiology of CSW is thought to be mediated by increased levels of atrial natriuretic peptide (ANP) and brain natriuretic peptide (BNP) which, are believed to be released after cerebral trauma [7-8]. ANP and BNP are also thought to directly inhibit mineralocorticoid production in the adrenal granulosa cells causing a hypo mineralocorticoid state [8]. CSW responds to fluid replacement and fludrocortisone which is a synthetic mineralocorticoid that binds cytosolic aldosterone and cortisol receptors [8-9].

## Case presentation

A previously healthy two-year-old male presented with two days of lethargy, progressive decrease in appetite, and emesis. On the day of admission, he developed altered mental status, loss of muscle tone, and perioral cyanosis. There was a concern for an unwitnessed seizure as the child appeared to be post-ictal on presentation to the emergency room where he was subsequently diagnosed with a large midline multi-cystic tumor producing obstructive hydrocephalus. Shortly after being transferred to the pediatric critical care unit (PICU), the child was noted to have unequal pupils with impending herniation. He was emergently endotracheally intubated and an external ventricular drain was placed with subsequent intraoperative cyst drainage. Magnetic resonance imaging (MRI) obtained when the child was stabilized revealed a large multi-cystic hypothalamic optic chiasm tumor. He underwent endoscopic cyst fenestration and subtotal resection of the mass. Pathology revealed low-grade juvenile pilocytic astrocytoma (WHO grade I). Pituitary function was evaluated pre-operatively and was relatively unremarkable, with normal thyroid function tests and a low random serum cortisol of 3.2 mcg/dL (2.9-17 mcg/dL). But the child was receiving high dose dexamethasone at the time. The peak cortisol level after stimulation with adrenocorticotropic hormone (ACTH) was normal at 21 mcg/dL.

**Figure 1:**
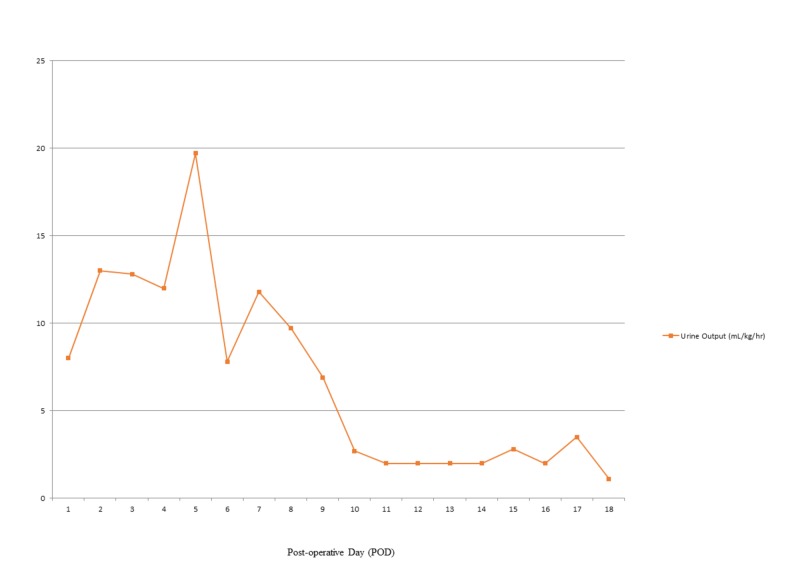
Effect of fludrocortisone initiated post-operative day nine on urine output

**Figure 2:**
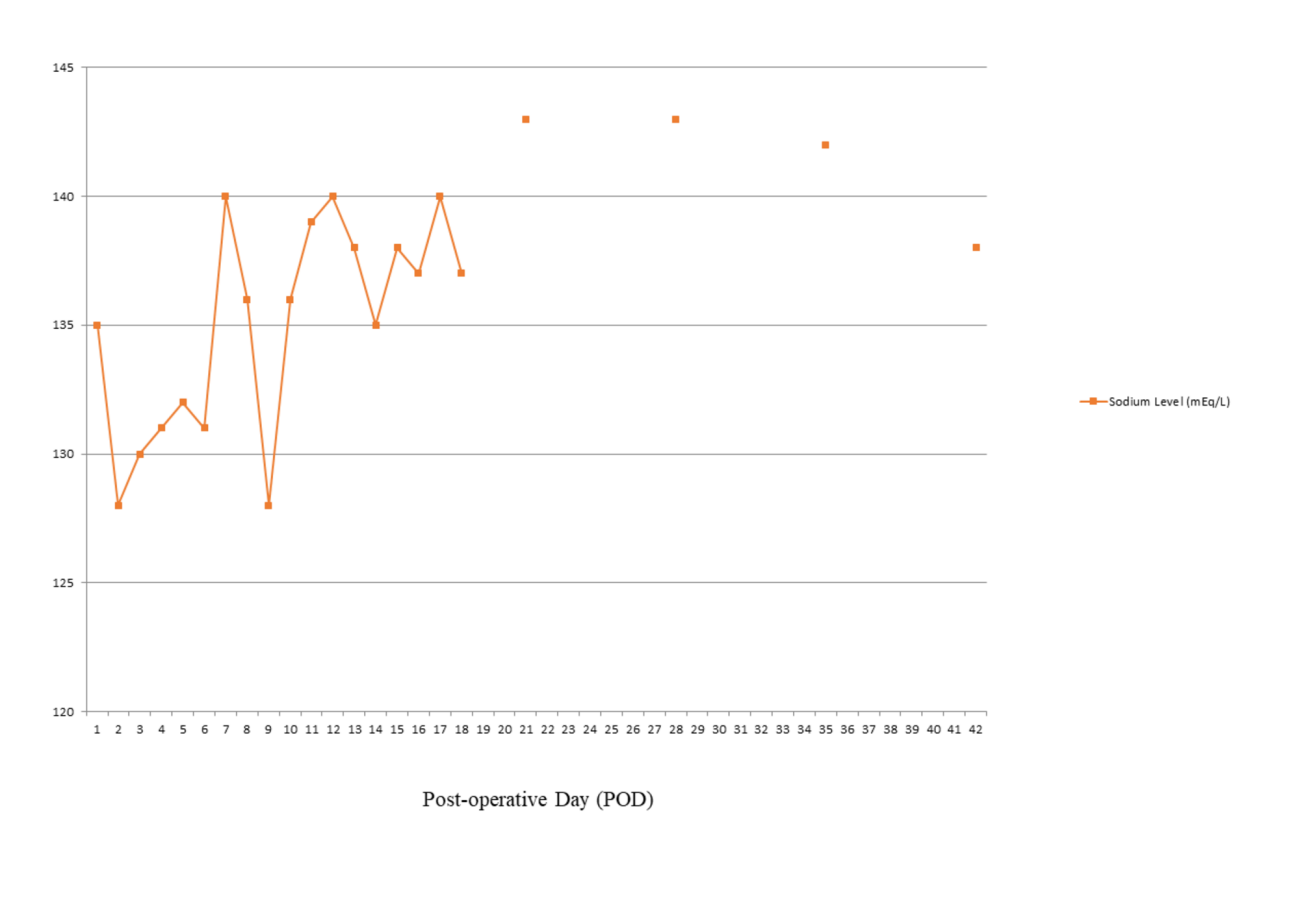
Effect of fludrocortisone initiated post-operative day nine on serum sodium level

On post-operative day two, the child was noted to develop polyuria and hyponatremia with urinary output as high as 13 mL/kg/hour and a steep decline in the serum sodium from 135 mEq/L to 128 mEq/L over six hours. Based on a very elevated urinary sodium excretion (229 mEq/L), with increased urine output (>4mL/kg/hr), elevated urine osmolality (523 mOsm/Kg) with a low serum osmolality (270 mOsm/Kg), and low uric acid (0.7 mg/dL), a diagnosis of CSW was made. The child required urine output replacement with isotonic fluids (Na 154 mEq/L) as well as the addition of 3% sodium chloride infusion as high as 3 mL/kg/hour to maintain a serum sodium of at least 130 mEq/L. On post-operative day six, enteral sodium replacement as high as 13 mEq/kg/day was initiated to facilitate weaning off the hypertonic saline. The hypertonic saline was discontinued but serum sodium values on post-operative day nine decreased to 128 mEq/L, demonstrating that the child could not maintain serum sodium levels off intravenous supplementation. The child was also unable to consistently tolerate the high enteral doses of sodium. Fludrocortisone 0.05 mg twice daily was initiated on post-operative day nine. Within 30 hours (after three doses) of treatment, polyuria declined, and serum sodium was maintained above 135 mEq/L (Figures 1, 2). Enteral sodium supplementation was successfully lowered to 3 mEq/kg/day. The child had no hypertension or hypokalemia associated with fludrocortisone administration. Once CSW was controlled, he underwent chemotherapy after subtotal resection of the tumor. Two months post-operatively; the child continues on fludrocortisone 0.05mg twice a day and oral sodium chloride at 3 mEq/kg/day with normal serum sodium and potassium levels. 

## Discussion

We present a case of a young child with refractory CSW that was successfully treated with fludrocortisone supplementation. This two-year-old child with a low-grade juvenile pilocytic astrocytoma (WHO grade I) developed significant hyponatremia on post-operative day two. He had associated polyuria and significant natriuresis, favoring the diagnosis of cerebral salt wasting syndrome over SIADH. He was then placed on large doses of parenteral and oral sodium chloride supplementation along with fluid replacement,. despite which his serum sodium levels did not normalize. He was also unable to tolerate such large oral doses of sodium chloride and was refusing to take them. These factors led to the initiation of fludrocortisone therapy. He responded very well to fludrocortisone treatment and is being maintained on it with reasonable amounts of oral sodium chloride supplementation which he is able to tolerate and with excellent results.

Sata et al. found that 22% of the patients that underwent transsphenoidal surgery for sellar tumors developed hyponatremia [1]. Patient fatality is reported to increase over tenfold if hyponatremia (<130 mEq/L) is present when compared to matched controls [3]. The two most common etiologies for non-iatrogenic hyponatremia, post-neurosurgical intervention, are CSW and SIADH [4]. It is very important to make an accurate diagnosis as the treatment of each of these conditions is very unique. Incorrect diagnosis of CSW may lead to inappropriate fluid restriction and may worsen the hypovolemia [5]. Additionally, two neurosurgical studies have found an increased risk of cerebral infarction with fluid restriction in patients with subarachnoid hemorrhage and hyponatremia (classically associated with CSW) [10-11]. CSW can cause severe and life-threatening hyponatremia and is generally managed with fluid replacement and sodium supplementation. The proposed pathophysiology of CSW is increased with the release of ANP and BNP secondary to cerebral trauma that results in natriuresis [6-7] and thus, the replacement of sodium losses is the mainstay of treatment. The two natriuretic peptides are also thought to inhibit the release of aldosterone via direct inhibitory effects on the zona glomerulosa of the adrenal cortex as well as having an indirect effect via the inhibition of renin release in the juxtaglomerular apparatus [8-9]. In the distal convoluted tubule, this increases the transcription of permeases and activates basolateral sodium-potassium adenosine triphosphatase (ATPase), both of which act to increase reabsorption of sodium into the circulation [7]. The role of fludrocortisone, a mineralocorticoid in the treatment of CSW is therefore physiologic and not surprising [12-13]. Our patient required very large doses of sodium supplementation to maintain his serum sodium at the lower end of the normal range. It was very challenging for this young child to tolerate such large amounts of salt by mouth. The addition of fludrocortisone overcame this challenge and has proven to be a successful treatment for our patient. Fludrocortisone use remains underexplored in the management of CSW at this age and should be considered especially in cases where conventional treatment fails or is challenging.

## Conclusions

In conclusion, based on our experience, we propose that mineralocorticoid supplementation is a safe and effective treatment of cerebral salt wasting syndrome. It may be considered as a first-line treatment, especially in severe or refractory cases of CSW.
